# A perspective on the development and lack of interchangeability of the breast cancer intrinsic subtypes

**DOI:** 10.1038/s41523-022-00451-9

**Published:** 2022-07-19

**Authors:** Francesco Schettini, Fara Brasó-Maristany, Nicole M. Kuderer, Aleix Prat

**Affiliations:** 1 Department of Medical Oncology, Hospital Clinic of Barcelona, Barcelona, Spain; 2 Translational Genomics and Targeted Therapies in Solid Tumors, August Pi I Sunyer Biomedical Research Institute (IDIBAPS), Barcelona, Spain; 3 Faculty of Medicine and Health Sciences, University of Barcelona, Barcelona, Spain; 4 Advanced Cancer Research Group, LLC, Kirkland, WA USA; 5 Breast Cancer Unit, IOB-QuirónSalud, Barcelona, Spain

**Keywords:** Cancer genomics, Breast cancer, Breast cancer, Biomarkers

Breast cancer intrinsic subtypes (IS) are biologically distinct entities, characterized by specific natural gene expression patterns^1,2^. The most widely accepted IS are the Luminal A, Luminal B, HER2-Enriched, and Basal-like^3^ (Fig. 1). These entities are prognostic and have potential therapeutic implications in both early-stage and advanced-stage hormone receptor-positive (HR+)/HER2-negative breast cancer^4–11^. However, the IS molecular classification is often misinterpreted, and immunohistochemistry (IHC)-based IS surrogates, or other molecular subtype definitions, are erroneously used interchangeably. This generates confusion for all the stakeholders involved, including scientists studying these biomarkers and physicians considering them for clinical decision-making. In this perspective, therefore, we provide readers with a historical overview of the discovery and clinical implementation of the IS, the main technical and biological differences among assays developed for their detection, and propose a specific and simple nomenclature for subtyping to avoid further confusion and disservice to patients.

**Fig. 1:**
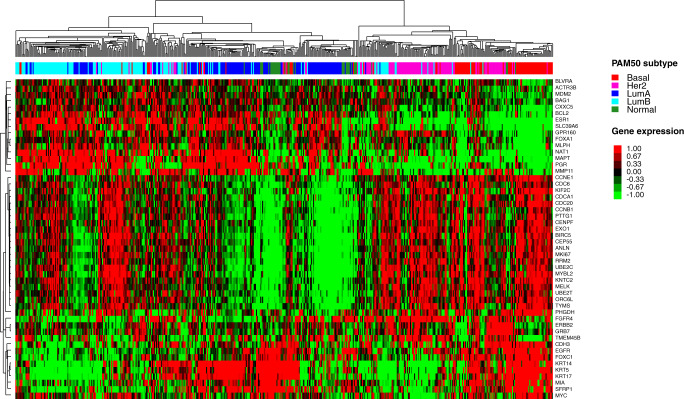
Heatplot representing the original, non-commercial PAM50 intrinsic subtypes’ genes, and gene expression patterns. Median centered unsupervised hierarchical clustering representing the breast cancer intrinsic subtypes (Luminal A, Luminal B, HER2-Enriched, Basal-like) and the Normal-like group, identified using the research-based PAM50 assay on the nCounter^®^ platform, in a set of 527 archived breast cancer fresh-frozen paraffin-embedded (FFPE) samples from Dr. Prat’s laboratory. Each column represents a single patient’s sample. The red color represents relatively high gene expression, green represents relatively low gene expression, and black represents median gene expression. The PAM50 gene list is reported on the right side of the heatplot. The unsupervised cluster and heatmap were obtained with R version 3.6.1, Cluster 3.0, and Javatreeview 1.1.6r4 for MacOSX. LumA luminal A, LumB luminal B, Her2 HER2-Enriched, Basal Basal-like, Normal Normal-like.

Breast cancer IS were first described in 2000 by Prof. Charles M. Perou and colleagues who utilized DNA microarrays representing more than 8000 genes in 65 breast tumor surgical specimens and 17 cultured cell lines^1^. Since then, several so-called intrinsic gene lists, methods, and platforms (e.g., Agilent, Affymetrix, and Illumina) for IS identification have been reported in the literature^2,3,12–17^. The intrinsic gene lists included hundreds of genes considered to reflect individual tumors’ phenotypes. However, several challenges prevented the implementation of IS in the clinic. First, most of the technologies applied required fresh-frozen tissue; second, the microarray technology requires resources and is time-consuming; third, the original classification based on hierarchical cluster analysis could only be applied retrospectively to sufficiently sized cohorts of patients; fourth, the entire sample-to-result process needed a centralized laboratory and a controlled environment.

These reasons led Perou and colleagues to develop a clinically applicable assay, which was first described in 2009^18^. First, they were able to shrink the number of intrinsic genes from almost 2000 to 50 (note: a lower number of genes was associated with reduced accuracy in subtype identification, especially for non-Basal-like subtypes). This gene list was called PAM50 (Fig. 1). Secondly, they used qRT-PCR to measure the expression of the 50 genes from readily available formalin-fixed paraffin-embedded tissue (FFPE), so to classify a patient’s tumor by assessing the similarities between a given case and prototypical IS centroids^18^. Furthermore, they derived a prognostic score known as the risk-of-relapse (ROR) score, capable of estimating a patient’s probability of breast cancer recurrence, by integrating and weighting the molecular subtype correlations, a subset of proliferation genes, and tumor size^18^. The standardized PAM50 qRT-PCR version was then implemented and commercialized. However, the standardization was not easy, and a central lab was needed. These issues were overcome when Nanostring Technologies^®^ developed the nCounter^®^ genomic platform, allowing for easy implementation of genomic assays through de-centralization, automated and fast process from RNA until the result, and, most importantly, without any enzymatic reaction. The PAM50 patent was licensed to Nanostring Technologies^®^, which re-developed the assay using the same genes (except the housekeeping genes used for normalization), standardized the test and created ad-hoc kits to allow for the decentralized processing of the assay with their platform^19,20^. The IS and ROR score from the Nanostring PAM50 decentralized assay (known as Prosigna^®^, which is now commercialized by Veracyte^™^) were CE-marked in Europe. Then, several trials prospectively confirmed the high reproducibility of the assay across labs and its impact on therapeutic decision-making in the context of early-stage HR + /HER2-negative breast cancer^21–23^. In the United States (US), the ROR score was FDA 510(k)-cleared but not IS; hence IS is not provided. Although IS are prognostic in early-stage HR+/HER2-negative disease, their independent clinical utility is not established in the presence of the ROR. The fact that IS are not reported by Prosigna^®^ in the US highlights the differences of obtaining FDA clearance, which contrasts with IS-related information provided by other commercial assays, which do not need FDA clearance if they are centrally performed as laboratory-developed tests (LDTs).

In 2010, a research-based study using microarray data showed that different single sample predictor (SSP) assays based on specific intrinsic gene lists presented only low to moderate IS concordance, with only Basal-like cancers consistently showing an almost-perfect agreement^24^. This led the authors to conclude that IS are inconsistent biomarkers that could not be incorporated into clinical practice^25^. Although none of the training sets and gene lists analyzed were specifically designed to be concordant at the individual-sample level, as we also highlighted elsewhere^24^, many research studies had used them interchangeably. At the same time, as also observed with a different approach by Haibe-Kains et al., a certain discordance remains across several subtype classifiers, including PAM50^26^. Again, the Basal-like subtype was consistently identified independently of the classifier used, while Luminal and HER2-Enriched subtypes were more difficult to classify^26^. An explanation of the discordance observed with several IS predictors in discriminating between Luminal subtypes is that the major biological difference between Luminal A and B tumors resides in their differential expression of proliferation-related genes, which exhibit a continuum of expression levels^26^. Hence different cut-offs distinguishing between a high and low proliferation level may impact on a single sample classification when different IS predictors are used. Conversely, the distinct features of the Basal-like subtype (i.e., high expression of basal cytokeratins, lack of expression of estrogen- and HER2-related genes)^27^ are likely responsible for the high levels of agreement in Basal-like identification among different IS classifiers.

In 2015, two investigators reported a microarray-based method, called AIMS, to identify IS of a single sample without using controls or references, showing a concordance with the original research-based PAM50 of 76–77%^28^. The authors highlighted the robustness of their assay and claimed to have solved normalization-related issues^28^. However, this approach has never been standardized, undergone regulatory review, nor commercialized. In parallel, another subtype assay was developed, standardized, and commercialized, namely the BluePrint^®^ test from Agendia^®^. Noteworthy, this subtype assay is not based on, and hence is not driven by, the natural IS gene expression patterns observed in the tumor. Instead, BluePrint^®^ took ER, PR, and HER2 IHC status as its starting point to develop gene expression patterns specifically able to discriminate between IHC hormone receptor-positive (HR+)/HER2-negative, HER2-positive (HER2+) and triple-negative breast cancer (TNBC)^29^. The gene profiles capable of identifying TNBC, HER2+and HR+/HER2-negative tumors were called Basal-type, HER2-type, and Luminal-type, respectively. Of note, the Luminal-type was further stratified by using the MammaPrint^®^ prognostic genomic assay, with the low-risk and high-risk cases being re-classified as Luminal A-type and Luminal B-type, respectively^29^. Hence, the BluePrint^®^ assay is a three-subtype classifier (derived from IHC-based prototypical groups), which uses the MammaPrint^®^ prognostic assay to further dissect HR+ tumors into a Luminal A and a Luminal B subgroup.

In the recent OPTIMA preliminary trial, where various commercial and standardized prognostic gene expression assays were compared, including PAM50/Prosigna^®^, the concordance of the results was low^30^. Importantly, the discordance rate between PAM50/Prosigna^®^ and BluePrint^®^ IS was 40%^30^. Such results are not surprising, since the two assays have different methodological approaches and gene lists (i.e., only seven genes in common among 130 genes)^18,29^. A similar challenge of lack of interchangeability is found even with assays based on a single antibody, such as with PD-L1 IHC clinical testing, where each assay is still unique due to differences in antibody performance characteristics or antigen scoring, and cannot be used interchangeably for indicating anti-PD1/PD-L1 therapy^31^.

Overall, these important differences in IS assays development, validation, and limited concordance lead us to the following considerations and recommendations:

1. The concept of molecular subtype is not comparable to the concept of a specific genetic mutation. The latter is a biologic feature that can be detected by different sequencing-based assays (with various levels of sensitivity). In contrast, each IS assay identifies different biologic entities, due to diverse gene expression construct assumptions and methodologies. Therefore, IS identified using distinct assays should be considered as different biomarkers trying to interrogate related but not identical biologic entities. In other words, IS-related assays are not interchangeable, unless a formal effort is made to develop fully concordant tests and a near-perfect agreement were to be observed, which unfortunately is not the case for the currently available assays^30^. This is an important misconception that currently exists in the medical community that we aim to clarify with this commentary (Fig. 2).

**Fig. 2:**
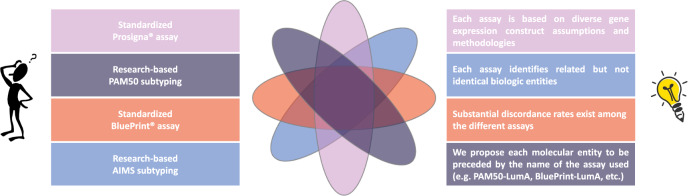
Main intrinsic/molecular subtypes classifiers and tips to avoid scientific and clinical confusion. Main intrinsic/molecular subtypes classifiers are resumed in the left boxes. In the right boxes, specific recommendations and a proposal for a new nomenclature of intrinsic/molecular subtypes are included, in order to avoid scientific and clinical confusion due to the lack of interchangeability between the assays. Diverse assays identify related but not identical biologic entities, which can result in different classifications of the intrinsic subtype for the individual patient, depending on the assay that is being used.
2. Standardization of gene expression data is possible and with high standards. However, we cannot assume that PAM50 research-based versions provide the same results as the standardized commercial assay (i.e., Prosigna^®^). In fact, researchers and physicians should understand a discordance rate of 10–20% between the formers and the latter (unpublished personal data, based on more than 10 years of experience performing the assays). This might be enough concordance to result in similar clinical utility, but without formally assessing both assays to prove this point, we cannot make such an assumption.
3. The fact that distinct assays use similar nomenclature to identify similarly but at the same time technically and biologically differing clinical entities (e.g., Luminal A and B established by different assays) is confusing for clinicians, patients, and researchers alike. Our proposal from now on is to clearly distinguish the name of the assay being used to identify IS, e.g., PAM50 subtypes (for research-based PAM50 analysis), Prosigna subtypes, BluePrint subtypes, AIMS subtypes, etc., instead of calling them all intrinsic subtypes, as if they were interchangeable biomarkers. Similarly, each subtype might be preceded by the name of the assay being used, e.g., PAM50-Luminal A, Prosigna-Luminal A, BluePrint-Luminal A, AIMS-Luminal A, etc. (Fig. 2).
4. Considering the absence of a perfect overlap between IHC-detected and genomic subtypes^9^, as well as the confusion observed in the literature regarding this aspect, we strongly encourage researchers and physicians to adopt the right terminology for IHC-based IS surrogates, which is the one proposed by the St. Gallen Breast Cancer International Consensus (i.e., Luminal A-like, Luminal B-like/HER2-positive or negative, HER2-positive, and Triple Negative)^32^.

Finally, an IS change from the primary to the recurrent/metastatic tumors has been detected in ~40% of the cases^33–35^. One reason might be the interference of a given treatment, resulting in reprogramming of gene expression patterns. Another cause could be a selective pressure exerted by a given therapy favoring the survival of resistant clones, likely reflecting underlying biology less sensitive to the treatment administered (e.g., Luminal A subtype shifting to HER2-Enriched subtype after prolonged endocrine therapy; HER2-Enriched subtype shifting to non-HER2-Enriched subtypes following anti-HER2-based treatments). Changes in subtype might be also due to natural tumor evolution and/or diverse organ microenvironment-specific influences on cancer gene expression^33–36^. Regardless, in all scenarios, the assays used for IS detection are the same. Therefore, in our view, there is no need for a further terminology change per se, as some have suggested.

## Reporting summary

Further information on research design is available in the Nature Research Reporting Summary linked to this article.

## Supplementary information

Reporting Summary
